# Performance Evaluation of Diagnostic Assays for Detection and Classification of Carbapenemase-Producing Organisms

**DOI:** 10.3390/antibiotics10121457

**Published:** 2021-11-26

**Authors:** Anru Zhang, Xiaojuan Wang, Xinyue Liang, Chaoe Zhou, Qi Wang, Jiangang Zhang, Hui Wang

**Affiliations:** Department of Clinical Laboratory, Peking University People’s Hospital, No. 11 Xizhimen South Street, Xicheng District, Beijing 100044, China; etoilann@163.com or curliele@163.com (X.W.); liangxinyuehh@sina.com (X.L.); zhouchaoe@163.com (C.Z.); wangqi_pkupph@163.com (Q.W.); zjg@bjmu.edu.cn (J.Z.)

**Keywords:** BD CPO detect panel, O.K.N detection kit, Rapidec Carba-NP, mCIM, eCIM, sCIM

## Abstract

Rapid and accurate detection can help optimize patient treatment and improve infection control against nosocomial carbapenemase-producing organisms (CPO). In this study, a total of 217 routine clinical isolates (*Enterobacterales* and *A. baumannii*), including 178 CPOs and 39 non-CPOs, were tested to evaluate the performance of six phenotypic carbapenemase detection and classification assays, i.e., BD Phoenix CPO detect panel, Rapidec Carba-NP, O.K.N detection kit, and three carbapenem inactivation methods (CIMs; mCIM, eCIM, sCIM). The overall detection sensitivity and specificity were 98.78% (95.21–99.79%) and 79.49% (63.06–90.13%), respectively, for the BD phoenix CPO P/N test; 91.93% (86.30–95.45%) and 100% (88.83–100%), respectively, for the Rapidec Carba-NP; 98.06% (94.00–99.50%) and 97.44% (84.92–99.87%), respectively, for mCIM; and 96.89% (92.52–98.85%) and 94.87% (81.37–99.11%), respectively, for sCIM. The classification sensitivity and specificity for the BD phoenix CPO Ambler test, the O.K.N detection kit, and the mCIM and eCIM were 56.71% (48.75–64.34%) and 94.87% (81.37–99.11%), 99.28% (95.43–99.96%) and 100% (88.83–100%), and 92.90% (87.35–96.23%) and 97.44% (84.92–99.87%), respectively. All detection assays were reliable in detecting carbapenemase. However, the Rapidec Carba-NP and mCIM were insufficient in detecting OXA-48-like enzymes. The BD phoenix CPO detect panel had a strong ability to detect carbapenemase but failed to classify 48/59 (81.36%) KPC, 8/52 (15.38%) NDM, 8/22 (36.36%) OXA-23-like, and 6/11 (54.55%) dual enzymes. The O.K.N detection kit accurately detected and differentiated KPC, NDM, and OXA-48-like enzymes existing alone or in combination. The results of this study will support reliable laboratory work tools and promote therapeutic and infection control decisions.

## 1. Introduction

Carbapenems are considered as the last resort to defend against severe multidrug-resistant Gram-negative bacterial infections owing to their broad spectrum of activity and stability to most β-lactamases [1]. Gram-negative organisms become carbapenems-resistant mainly through carbapenemase production, cephalosporinase (AmpC), and/or extended-spectrum β-lactamase (ESBL) expression in combination with porin-encoding genes (such as oprD) mutation and/or efflux pump (such as MexAB-OprM, MexXY-OprM, MexCD-OprJ) overexpression, which lead to limited cell membrane permeability [2,3,4,5]. Carbapenemase, a primary mechanism of carbapenem resistance in Gram-negative bacteria, is mainly encoded on plasmids and is highly transmissible [6]. Over the past decades, carbapenem-producing organisms (CPOs) have become increasingly prevalent worldwide, and infections caused by carbapenem-resistant organisms, especially CPOs, are associated with limited clinical treatment options, a high mortality rate, and a heavy healthcare burden.

Early screening and identification of carbapenem-producing organisms (CPO), at clinical infections or the carriage state, is efficient to prevent the development of hospital outbreaks and to instruct clinical treatment and intervention of these severe multidrug-resistant bacteria infections. Phenotypic detection assays, including chromogenic media, the Carba-NP test [7], carbapenem inactivation method (CIM) [8], and matrix-assisted laser desorption ionization–time of flight mass spectrometry [9,10] analysis, are widely used to detect carbapenemases in clinical isolates, but cannot accurately classify the enzymes produced by the isolates. Commercial diagnostic kits based on immunochromatography principle, such as the OXA-48 K-Se T [11], O.K.N K-Se T [12], and CARBA-5 reagents [13], have high sensitivity and specificity. Genetic detection methods are considered to be the gold standard, such as traditional PCR, Xpert Carba-R, reverse transcription quantitative PCR, multiplex real-time PCR, and the microfluidic chip technology, which have high sensitivity and specificity, and can directly identify carbapenemase genes. However, the high cost and the requirement of specialized instruments and skilled staff limited their application for many laboratories worldwide without such resources [14,15,16]. Moreover, PCR-based methods may have false positives and false negatives, and may miss new variant types of carbapenemase.

To improve carbapenemase detection and classification, this study evaluated the performance of three commercial diagnostic kits—BD Phoenix CPO detect panel, Rapidec Carba-NP, and an O.K.N detection kit—and three carbapenem inactivation methods (CIMs)—mCIM, eCIM, and the simplified version sCIM—using clinical isolates collected in China.

## 2. Results

### 2.1. General Characteristics of the Tested Strains

A total of 217 clinical isolates were included in this study (*A. baumannii*, *n* = 23; *E. coli*, *n* = 41; *Enterobacter cloacae*, *n* = 20; *Klebsiella oxytoca*, *n* = 5; *Klebsiella aerogenes*, *n* = 9; *K. pneumoniae*, *n* = 102 and 17 other *Enterobacterales* species). Only one isolate per species of one patient was included. By genetic characterization, there were 178 isolates carry carbapenemase genes (59 class A carbapenemase (KPC, *n* = 59), 69 class B carbapenemase (NDM, *n* = 52; IMP, *n* = 15; VIM, *n* = 1; SIM, *n* = 1), 39 class D carbapenemase (OXA-23-like, *n* = 22; OXA-58-like, *n* = 1; OXA-48-like, *n* = 16), and 11 dual carbapenemase (KPC + NDM, *n* = 4; KPC + IMP, *n* = 2; NDM + IMP, *n* = 6)) (Table 1). 

### 2.2. Overall Performance

The sensitivity and specificity of the various assays for carbapenemase detection and classification are shown in Table 2. The BD phoenix CPO detect P/N test, mCIM, and sCIM had sensitivity of over 96% in detecting the carbapenemase production. The Rapidec Carba-NP had high specificity of 100%, but the sensitivity was 91.93% due to its low efficiency in detecting OXA-48-like carbapenemase. The O.K.N detection kit had high sensitivity and specificity of over 99% and 100% in detecting and classifying the carbapenemase within its detection range.

### 2.3. Accuracy of Assays in Detecting and Classifying Specific Carbapenemase

A total of 164 CPOs and 39 non-CPOs were tested by the BD Phoenix CPO detect panel. Compared to the molecular testing, the P/N test detected 162/164 (98.78%) CPOs and incorrectly detected 8/39 (20.51%) carbapenemase-negative isolates as carbapenemase producers (specificity, 79.49%). The Ambler test provided a correct Ambler classification of 93/164 (56.71%) CPOs, as well as 63/164 (38.41%) unclassified and 8/164 (4.88%) misclassified. Five of sixty-nine (7.25%) class B carbapenemase producers were misclassified as a class D producer (NDM-carrier, *n* = 4; IMP-carrier, *n* = 1) and three OXA-23-carrying *A. baumannii* (3/22, 13.64%) were misclassified as a class B producer. Furthermore, 48/63 (76.19%) KPC, 4/63 (6.35%) NDM producer, and 5/63 (7.94%) *A. baumannii* with OXA-23-like were not classified. Eleven isolates with more than one carbapenemases-encoding gene were included in this study; however, only 5/11 (45.45%) had been classified as producing one type of carbapenemase, and 5/6 (83.33%) of the heterotypic carbapenemase (class A + B producer) cannot be distinguished (Table 3).

The performance of the Rapidec Carba-NP test, the O.K.N detection kit, and three carbapenem inactivation methods (mCIM, sCIM, mCIM, and eCIM) was evaluated on 161 and 39 routine strains with and without CPO suspicion. The carbapenemase detection sensitivity and specificity were 91.93% and 100%, 98.06% and 97.44%, and 96.86% and 94.87% for the Rapidec Carba-NP test, mCIM, and sCIM, respectively. The Rapidec Carba-NP had detected all 128 class A and class B enzymes but failed to identify 11/16 (68.75%) OXA-48-like enzyme and 2 strains with the KPC + IMP enzyme. The mCIM seemed insufficient in detecting OXA-48-like enzymes, and the sCIM seemed insufficient in detecting Acinetobacter baumannii producing OXA-23-like enzyme. The O.K.N detection kit could detect and classify almost all strains producing carbapenemase within its test range for its high sensitivity (99.28%) and specificity (100%). The mCIM and eCIM, with classification sensitivity and specificity of 92.90% and 97.44%, respectively, failed to classify 4/59 (6.78%) KPC enzymes, 3/15 (20.00%) IMP enzymes, 4/16 (25.00%) OXA-48-like enzymes, and could only report serine carbapenemase among 6 class A + B enzyme producers (Table 4).

## 3. Discussion

Rapid and accurate detection and classification of CPOs is critical in avoiding treatment failures and guiding clinical antibiotics therapy and infection control decisions, including the use of expensive antibiotics and isolation measures. However, phenotypic identification of organisms with mu ltiple carbapenemase types, low-level carbapenemase expression, extended-spectrum beta-lactamases (ESBLs), AmpC beta-lactamases, growth impairment, and/or other potentially uncovered carbapenemase resistance mechanisms can be difficult [17].

The BD Phoenix CPO detect panel offers (semi)-automated antimicrobial susceptibility testing (AST) systems and phenotypic detection and classification of carbapenemase [18]. The high sensitivity of the BD Phoenix CPO detect panel P/N test observed in this study supports the direct reporting of CPO-negative strains without additional tests, and directly provides the true carbapenem MICs that can be used as a screening value for carbapenemase. However, the limitation was classification performance, especially in typing class A β-lactamase, with sensitivity of only 18.64%, which corresponds with the results of Simon et al. (5/13, 38.46%) [19] and Ong et al. (13/30, 43.33%) [20], but was much lower than that reported by Thomson et al. (91/110, 82.73%) [21]. Eleven strains producing dual carbapenemases were determined as positive by the P/N test but without providing an Ambler classification or with attributing only one of the involved carbapenemases by the Ambler test. It is also noteworthy that 8 of 39 (20.51%) non-CPOs were false positive, which indicated downstream tests, such as a colorimetric assay and/or CIM tests, were needed to confirm the positive result detect by the BD Phoenix CPO detect P/N test. The performance of the BD Phoenix CPO detect P/N test could be challenged by the non-CPOs phenotypes, with decreased efficiency and specificity in detecting strains combining ESBLs and/or AmpCs with/without porin loss [21,22,23]. Among the eight false positive strains, 2/8 were AmpC producers, 3/8 were ESBLs producers, and 2/8 harbored both AmpC beta-lactamases and ESBLs.

Interpretation the result of the Rapidec Carba-NP can be challenging for an inexperienced operator, and inconclusive results can occur. Moreover, the detection of carbapenemases in mucoid isolates and the detection of OXA-48-like carbapenemase could be challenging, which was consistent with several previously published studies [24,25,26].

The O.K.N detection kit, a method with no special equipment and professionals required, interpretate easily and clearly, and could detect KPC, NDM, and OXA-48-like enzymes and their combination efficiently and rapidly. However, IMP (IMP-4, IMP-26), which is referred to as one of “the big five” and had prevalence in China [27], could not be identified. The recently launched Carba5 system [13], the RESIST-4 O.K.N.V. assay [28], and the RESIST-5 O.K.N.V.I. assay [29] could perform a multiple lateral flow immunoassay on NDM, KPC, IMP, VIM, and OXA-48-like enzymes, and can detect multiple enzymes produced by strains.

The mCIM and eCIM, recommended by CLSI guidelines for their high sensitivity and specificity in the detection of KPC, NDM, VIM, IMP, IMI, SPM, SME, and OXA enzymes carried by *Enterobacterales* bacteria [30], could tell serine β-lactamase (SBL) and metallo-β-lactamase (MBL), which will affect the clinical therapy decisions, such as the use of ceftazidime-avibactam [28]. However, it seemed insufficient when classified IMP and OXA-48-like enzyme. The sCIM test was an easy, rapid, efficient, and cheap method, but the results might be influenced by the amount of bacteria on the imipenem disk, since the result was negative with a small bacterial sample, but could become “uncertain” with more abundant bacteria in the tested sample.

## 4. Methods

### 4.1. Bacterial Strains

A total of 217 routine clinical isolates was tested to evaluate the performance of six phenotypic carbapenemase detection and classification assays, i.e., BD Phoenix CPO detect panel, Rapidec Carba-NP, an O.K.N detection kit, and three carbapenem inactivation methods (CIMs) (mCIM, eCIM, and sCIM). These isolates include 178 CPOs and 39 non-CPOs, the carbapenemase activity of which was previously tested by the mCIM and eCIM test according to CLSI guidelines [30], and then genetically confirmed by traditional PCR and sequencing. The primers used to amplify specific carbapenenmase genes are shown in Table 5. Strains collected from 66 general hospitals in 23 provinces of China from 2011 to 2018 were stored at −70 °C and incubated overnight on Columbia agar plate with 5% sheep blood at 35 ± 1 °C. To ensure the purity of strains, sub-incubation was carried out. Identification was conducted by matrix-assisted laser desorption ionization–time of flight mass spectrometry (Bruker Daltonics, Bremen, Germany) using α-cyano-4-hydroxycinnamic acid matrix and standard protocol. Species identification was determined by scores > 2.

### 4.2. BD Phoenix CPO Detect Panel

The BD Phoenix CPO detect panel (Becton, Dickinson and Company, Sparks, MD, USA) is a growth-based qualitative confirmatory method (ID/NMIC 503 panel, NMIC 502 panel), which could identify species and use drugs such as carbapenems and β-lactams, alone and in combination with various chelators and β-lactamase inhibitors required for CPO detection and classification to detect and confirm A, B, and D carbapenemase [17]. In the CPO detect panel, the ID/NMIC 503 panel aimed to identify species and detect carbapenemase activity (P/N test), and the NMIC 502 panel aimed to identify carbapenemase producers according to the Ambler classification (Ambler test). Not all isolates determined carbapenemase positive by the P/N test could be classified by the Ambler test. The P/N test-negative results with Ambler classification were previously demonstrated to be impossible [21]. Strains of 141 carbapenemase-producing *Enterobacterales* (CPE), 23 carbapenemase-producing *Acinetobacter baumannii* (CPAB), and 39 non-CPOs were incubated overnight using the BD M50 system, and results were interpreted by Epi-Centre software.

### 4.3. Rapidec Carba-NP Test

The Rapidec Carba-NP kit (bioMérieux, La Balme-les-Grottes, France) is a rapid biochemical test based on detecting the hydrolysis of the β-lactam ring of imipenem. The test was performed in accordance with the manufacturer’s recommendations [31]. A full loop (10 μL) of bacterial colonies from a culture plate was mixed into the well c so that the turbidity is similar to well b. Visual observations then occurred after 30 min incubation at 37 °C and, if necessary, after 2 h. A color change from red-to-yellow or red-to-orange was considered positive. The visual assessment and results interpretation were conducted blindly by two technicians who had no information about carbapenemase production of these strains.

### 4.4. O.K.N Detection Kit

Then, 200 isolates were tested by O.K.N Detection kit (Beijing Gold Mountainriver Tech Development Co., Ltd., Beijing, China). A single colony on blood agar plate was suspended in 10 drops of lysis solution, and then three drops of the diluted sample were added onto the test strip. The results were read within 15 min with naked eyes according to the synopsis.

### 4.5. Carbapenem Inactivation Methods

The mCIM and eCIM tests were performed following the protocol recommended by the Clinical Laboratory Standards Institute (CLSI) guidelines [30]. In the sCIM test [32], one side of the imipenem disk was smeared with the tested strain instead of soaked in tryptic soy broth (TSB) containing the strain for 4 h, and then placed on the MHA plate inoculated with meropenem-susceptible *Escherichia coli* ATCC 25922.

### 4.6. Quality Control

*K. pneumoniae* BAA-1705 (producing KPC), *E. coli* ATCC-2452 (producing NDM), and *E. coli* BAA-2523 (producing OXA-48) were used as positive quality control strains, whereas *K. pneumoniae* ATCC 700603 was used as a negative quality control in evaluation of the BD Phoenix CPO detection panel. In the CIM tests, *E. coli* ATCC 25922 and *K. pneumoniae* BAA 1705 were used as negative and positive quality control strains, respectively.

### 4.7. Statistical Analysis

The sensitivity and specificity of each method were analyzed along with their 95% confidence intervals, and compared with the results from reference standard molecular characterization using the free software VassarStats (http://vassarstats.net/; 28 September 2021).

## 5. Conclusions

Taken together, the results of this study suggest that the BD Phoenix CPO detect P/N test, mCIM and sCIM were reliable for CPOs screening and detecting. However, due to the relatively low detection specificity and low classification sensitivity, additional tests and further improvements are required to confirm positive detection by the BD Phoenix CPO detect panel P/N test and to improve the performance of the Ambler test. Moreover, when the result of Rapidec Carba-NP was interpreted negative, additional tests such as mCIM and sCIM were needed to exclude the OXA-48-like enzyme. The O.K.N detection kit with high classification efficiency indicates a rapid, easy and reliable tool to classify KPC, NDM, and OXA-48-like enzymes.

## Figures and Tables

**Table 1:** Overview of strains included in this study.

Ambler Class	Carbapenemase	*A. baumannii*	*Citrobacter freundii*	*E. cloacae*	*E. coli*	*Klebsiella aerogenes*	*Klebsiella oxytoca*	*K. pneumoniae*	*Serratia marcescens*	Others
ClassA	KPC		1	1	3	1		52		1
ClassB	NDM		1	7	25	2	2	11	1	3
	IMP		5	5	2			3		
	VIM							1		
	SIM							1		
ClassD	OXA-23	22								
	OXA-58	1								
	OXA-48-like							16		
Dual enzymes	KPC + NDM				1			3		
	KPC + IMP					2				
	NDM + IMP			2			2	1		
non-CPO			2	5	10	4	1	14	2	1
Total		23	9	20	41	9	5	102	3	5

KPC, *Klebsiella pneumoniae* carbapenemase; NDM, New Delhi metallo-β-lactamase; IMP, imipenemase; VIM, Verona integron-encoded metallo-β-lactamase; SIM, SIM-type metallo-β-lactamases; OXA, oxacillinase.

**Table 2:** Overall sensitivity and specificity of phenotype diagnostic assays.

Diagnostic Assays		Sensitivity		Specificity	
		%	95% CI	%	95% CI
BD Phoenix CPO detect panel	P/N test	98.78	95.21–99.79	79.49	63.06–90.13
	Ambler test	56.71	48.75–64.34	94.87	81.37–99.11
CPO detection tests	Rapidec Carba-NP	91.93	86.30–95.45	100	88.83–100
	mCIM	98.06	94.00–99.50	97.44	84.92–99.87
	sCIM	96.89	92.52–98.85	94.87	81.37–99.11
CPO classification tests	O.K.N Detection kit	99.28	95.43–99.96	100	88.83–100
	mCIM + eCIM	92.90	87.35–96.23	97.44	84.92–99.87

**Table 3:** Performance of BD Phoenix CPO detect panel.

Ambler Class	Carbapenenmase	BD Phoenix CPO P/N Test	BD Phoenix CPO Ambler Test			
			ClassA	ClassB	ClassD	Unclassfied
ClassA						
	KPC (*n* = 59)	58	11			48
ClassB						
	NDM (*n* = 52)	52		44	4	4
	IMP (*n* = 15)	15		14	1	
	VIM (*n* = 1)	1		1		
	SIM (*n* = 1)	1		1		
ClassD						
	OXA-23 (*n* = 22)	21		3	14	5
	OXA-58 (*n* = 1)	1			1	
	OXA-48-like (*n* = 2)	2			2	
Dual enzymes						
Class A + B	KPC + NDM (*n* = 4)	4				4
	KPC + IMP (*n* = 2)	2	1			1
Class B + B	NDM + IMP (*n* = 5)	5		4		1
non-CPO	(*n* = 39)	8	1	2		5

**Table 4:** Performance of other carbapenemase detection and classification diagnostic assays.

Ambler Class	Carbapenemase	CPO Detection Tests			CPO Classification Tests	
		Rapidec Carba-NP	mCIM	sCIM	O.K.N Detection Kit	mCIM + eCIM
ClassA						
	KPC (*n* = 59)	59	59	58	59	55
ClassB						
	NDM (*n* = 52)	52	52	52	51	52
	IMP (*n* = 15)	15	15	15	0	12
	VIM (*n* = 1)	1	1	1	0	1
	SIM (*n* = 1)	1	1	1	0	1
ClassD						
	OXA-23 (*n* = 5)	5	/	1	0	/
	OXA-58 (*n* = 1)	1	/	1	0	/
	OXA-48-like (*n* = 16)	5	13	16	16	12
Dual enzymes						
Class A + B	KPC + NDM (*n* = 4)	4	4	4	4	4
	KPC + IMP (*n* = 2)	0	2	2	2	2
Class B + B	NDM + IMP (*n* = 5)	5	5	5	5	5
non-CPO	(*n* = 39)	0	1	2	0	1

**Table 5:** Primers used in this study.

Primer Name	Targeting Gene	Nucleotide Sequence	Product Size (bp)
KPC-F	*bla*_KPC_	5′-TGTCACTGTATCGCCGTC-3′	1010
KPC-R		5′-CTCAGTGCTCTACAGAAAACC-3′	
NDM-F	*bla*_NDM_	5′-ATGGAATTGCCCAATATTATGCAC-3′	813
NDM-R		5′-TCAGCGCAGCTTGTCGGC-3′	
IMP-F	*bla*_IMP_	5′-TGAGCAAGTTATCTGTATTC-3′	740
IMP-R		5′-TTAGTTGCTTGGTTTTGATG-3′	
VIM-F	*bla*_VIM_	5′-TTATGGAGCAGCAACCGATGT-3′	920
VIM-R		5′-CAAAAGTCCCGCTCCAACGA-3′	
SIM-F	*bla*_SIM_	5′-TACAAGGGATTCGGCATCG-3′	571
SIM-R		5′-TAATGGCCTGTTCCCATGTG-3′	
OXA-48-like-F	*bla*_OXA-48-like_	5′-GCGTGGTTAAGGATGAACAC-3′	438
OXA-48-like-R		5′-CATCAAGTTCAACCCAACCG-3′	
OXA-23-like-F	*bla*_OXA-23-like_	5′-GATCGGATTGGAGAACCAGA-3′	501
OXA-23-like-R		5′-ATTTCTGACCGCATTTCCAT-3′	
OXA-58-like-F	*bla*_OXA-58-like_	5′-AAGTATTGGGGCTTGTGCTG-3′	353
OXA-58-like-R		5′-CCCCTCTGCGCTCTACATAC-3′	

## Data Availability

Not applicable.
